# Cooperation and Education

**DOI:** 10.1055/a-2642-2254

**Published:** 2025-07-23

**Authors:** Alberto Musolas

**Affiliations:** 1 Department of Plastic, Aesthetic and Reconstructive Surgery, Plastic Surgery Unit, Centro Médico Augusta, Barcelona, Spain

It was probably in May or June of 1984 when the boss asked for someone to go to work in Africa. Nobody answered. He then specified, “In a very small hospital in the forest of Cameroon.” That was my chance to get involved in adventures in the forest, so I volunteered. My initiation into cooperation was not particularly “humanitarian,” but the reality of what I found in Logbikoy in 1984—the people, their needs, their suffering, and also their joy and resilience—captivated me for the rest of my life. I became more of a cooperator than an adventurer.

However, cooperation is, and should be, more than simply helping others.

The phrase “help kills help,” attributed to Mr. Sankara, the former president of Burkina Faso, means, in other words, that cooperation should contribute to making itself unnecessary in the long run.

Thus, cooperation should include education to empower the people being helped so that they can eventually do on their own what is currently done by cooperators.

In this sense, with my friends Javier Beut, Fernando Fonseca (+), and especially Pierre Quinodoz, we moved from “assisting missions,” which are highly beneficial for individuals, to “assisting-educating missions,” which are highly beneficial for society as a whole as well as for individuals.

Plastic surgery is generally considered by our society to be a luxury, a profitable specialty in medicine, and thus people rarely associate it with cooperation.

Beyond providing solutions for the majority of defects and pathologies of each patient, great efforts must be made to transmit our knowledge to the young native surgeons working in the field. However, teaching and education should be adapted to the conditions they encounter daily. This also requires humility.

In developing countries, secure and reliable procedures that can be performed under limited or very limited resources should be prioritized over sophisticated and complex ones that are usually beyond the reach of local surgeons.

However, the reality on the ground is terrifying. Congenital malformations, burn sequelae, tumors, war injuries, and, more recently, open fractures and injuries resulting from traffic accidents are shocking realities in developing countries. Plastic surgery is now an essential specialty in all of them. It is a crucial part of the public health system because it has the capacity to reintegrate disabled patients into society. In the words of the Indian surgeon Dr. Ballah Subbaiah, “Plastic surgery is the branch of Medicine that truly challenges Nature's verdict. It gives a person the possibility of a renaissance, and in our context, India, it provides the opportunity for an in-life reincarnation as it allows the patient to initiate, without its defect, a new human life with dignity.”

Backed by 49 humanitarian missions over 41 years, two things have become clear to me: Cooperation and education are debts owed by those who have access to everything to those who do not. Additionally, plastic surgery is an essential and much-needed specialty in developing countries.

